# Kinect v2-Assisted Semi-Automated Method to Assess Upper Limb Motor Performance in Children

**DOI:** 10.3390/s22062258

**Published:** 2022-03-15

**Authors:** Celia Francisco-Martínez, José A. Padilla-Medina, Juan Prado-Olivarez, Francisco J. Pérez-Pinal, Alejandro I. Barranco-Gutiérrez, Juan J. Martínez-Nolasco

**Affiliations:** 1 Electronics Engineering Department, National Technology of Mexico in Celaya, Celaya 38010, Mexico; d1903003@itcelaya.edu.mx (C.F.-M.); alfredo.padilla@itcelaya.edu.mx (J.A.P.-M.); francisco.perez@itcelaya.edu.mx (F.J.P.-P.); israel.barranco@itcelaya.edu.mx (A.I.B.-G.); 2 Mechatronics Engineering Department, National Technology of Mexico in Celaya, Celaya 38010, Mexico; juan.martinez@itcelaya.edu.mx

**Keywords:** Kinect v2, Fugl-Meyer, upper limb, measurement, COVID-19

## Abstract

The interruption of rehabilitation activities caused by the COVID-19 lockdown has significant health negative consequences for the population with physical disabilities. Thus, measuring the range of motion (ROM) using remotely taken photographs, which are then sent to specialists for formal assessment, has been recommended. Currently, low-cost Kinect motion capture sensors with a natural user interface are the most feasible implementations for upper limb motion analysis. An active range of motion (AROM) measuring system based on a Kinect v2 sensor for upper limb motion analysis using Fugl-Meyer Assessment (FMA) scoring is described in this paper. Two test groups of children, each having eighteen participants, were analyzed in the experimental stage, where upper limbs’ AROM and motor performance were assessed using FMA. Participants in the control group (mean age of 7.83 ± 2.54 years) had no cognitive impairment or upper limb musculoskeletal problems. The study test group comprised children aged 8.28 ± 2.32 years with spastic hemiparesis. A total of 30 samples of elbow flexion and 30 samples of shoulder abduction of both limbs for each participant were analyzed using the Kinect v2 sensor at 30 Hz. In both upper limbs, no significant differences (*p* < 0.05) in the measured angles and FMA assessments were observed between those obtained using the described Kinect v2-based system and those obtained directly using a universal goniometer. The measurement error achieved by the proposed system was less than ±1° compared to the specialist’s measurements. According to the obtained results, the developed measuring system is a good alternative and an effective tool for FMA assessment of AROM and motor performance of upper limbs, while avoiding direct contact in both healthy children and children with spastic hemiparesis.

## 1. Introduction

Effective public health and social measures are fundamental strategies to combat Coronavirus (COVID-19). Avoiding crowds and maintaining a physical distance from others is essential to reduce the rate of infection [1]. The negative consequences of the interruption of rehabilitation activities are significant for the population with physical disabilities [2]. The World Health Organization reported that more than 15% of the world’s population has a physical limitation, such as cerebral palsy (CP) [3]. The Gross Motor Function Classification System (GMFCS) scale is essential for determining physical movement skills in patients with CP, e.g., the ability to sit up [4]. Similarly, the Manual Ability Classification System (MACS) scale determines fine motor skills, specifically of the hand, e.g., activities that the patient can perform daily, from combing hair to tying shoelaces [5]. Motor treatment is essential, and the effectiveness of cerebral palsy treatment depends on many factors, including optimal and systematic monitoring of patients’ progress [6]. Home therapy with active video game technologies and low-cost virtual reality is a favorable solution to minimize the impact of rehabilitation interruptions [2].

Recently, telemedicine has increased exponentially due to the COVID-19 pandemic. The authors Ben-Pazi et al. [7], in their various recommendations, mention the possibility of a range of motion (ROM) measurement based on photographs of the patient that are remotely taken and sent to specialists. Clinical ROM measurements are essential metrics for monitoring motor rehabilitation progress. In general terms, this provides objective and reliable information in the diagnosis and clinical monitoring of the physical condition evolution [8]. The goniometer is one of the most common instruments for measuring ROM [9]. Sophisticated instruments are also used such as: electro-goniometers [10], laser goniometers [11], optoelectronic devices [12], wearable sensors [13,14], smartphone applications [15], Kinect sensors [16], and Xsens systems [17].

In this context, the Kinect v2 sensor (Microsoft, Redmond, WA, USA) has a color camera and a depth measurement system based on active illumination (infrared camera and an infrared projector) [18,19]. Hence, color, depth, and infrared image detection are possible, which turns the Kinect into a low-cost three-dimensional RGB-D camera [20]. Time-of-Flight depth-sensing technology allows object distance measurements for each image pixel according to the output data. Depth maps correspond to 16-bit encoded 2D images, containing the measurement information stored in a pixel array; from this 2D data, it is possible to infer 3D coordinates (X, Y, Z) [18]. The natural user interface (NUI) presents a markerless human pose estimation algorithm; its main objective is interpreting human positions and gestures by processing vector data.

Several researchers have evaluated the reliability of the Kinect v2 system in the analysis of upper limb functionality and found significant measurement accuracy [16,21,22,23,24,25,26,27]. The authors Cai et al. [21] found that the mean square error of the shoulder joint angle (adduction and abduction) was less than six degrees between the Kinect v2 (Microsoft, Redmond, WA, USA) and Vicon (Oxford Metrics Group, Vicon Motion Systems Ltd., Oxford, UK). After a stroke, the FMA scale is used to assess the motor performance of the upper limb. This scale is composed of an assessment of motor and sensory abilities, balance, joint range of motion, and joint pain [28]. For example, the Kinect sensor has been used in FMA tests, mainly because it allows the measurement of ROM [29,30,31,32]. The authors Kim et al. [30] found a significant correlation between FMA scores and scores using the Kinect v1 sensor (Microsoft, Redmond, WA, USA). They claim that the use of FMA with Kinect v1 is a reliable way to assess upper limb motor performance. Recently, Lee et al. [33] proposed an FMA scoring algorithm with fuzzy logic and Kinect v2; the authors argue that this method presents a high correlation with the scores given by a specialist.

A comparison of upper limbs’ angular measurements using a Kinect v2 sensor (Microsoft, Redmond, WA, USA) and a universal goniometer (Richardson Products Inc, Gulfstream Rd, Frankfort, KY, USA) was undertaken in the study described in this paper. In addition, a semi-automated FMA clinical scoring algorithm was implemented. The main study goal was to assess upper limbs’ motor performance using the proposed semi-automated method assisted by the Kinect v2 sensor, from which the results were contrasted with specialist’s measurements.

## 2. Materials and Methods

An experimental study using a low-cost natural user interface (NUI) system to quantitatively measure AROM and clinically FMA assess the upper limb motor skills in children was conducted. Firstly, the measuring system were implemented using the selected components. Then the designed graphical user interface was programmed and finally validated in the experimental stage.

### 2.1. Participants

Two test groups, each having eighteen participant children, were analyzed in the experimental stage, where upper limbs’ AROM was measured and motor performance was assessed using FMA. In the control group, participants (mean age of 7.83 ± 2.54 years) had no cognitive impairment or upper limb musculoskeletal problems. The study test group comprised children aged 8.28 ± 2.32 years with spastic hemiparesis. A total of 30 samples of elbow flexion and 30 samples of shoulder abduction of both limbs for each participant were analyzed using the Kinect v2 sensor. In addition, 30 FMA assessments tests were performed [28]. All participants gave informed consent. The inclusion and rejection criteria for both groups are presented in Table 1.

#### Measurement Protocol

The participant remained seated and performed a voluntary movement within the flexor synergies according to the FMA scoring; Fugl-Meyer (1975) [28]. The measurement conditions were the same as those of the preliminary test (Section 2.3).

A physiotherapist performed upper limbs’ AROM measurement with a goniometer and motor performance assessment using FMA scoring. The cognitive characteristics for each participant were assessed by a psychologist using the Bender Koppitz test [34]. It is relevant to mention that, of the sample of 27 candidates initially participating in the study group, nine were excluded: three who did not want to participate in the evaluations, and six due to severe mental age lags detected by the Bender Koppitz test [34]. However, following the psychologist’s suggestion, participants with mental age deficits capable of following directions were accepted as participants in the study because, in most cases, the chronological mismatch was due to hand manipulation problems in performing the activities required by the test (Table 2).

### 2.2. Graphical User Interface and Programming

A Kinect v2 sensor and an HP Intel^®^ Core™ i7-9750H 9750H 2.60 GHz laptop with NVIDIA GeForce GTX 1650 graphics card was used to integrate the AROM measurement and FMA assessment system. The Kinect v2 sensor (Microsoft, Redmond, WA, USA) was employed to assess children’s limb movements based on angles formed by the limb joints.

**Figure 1:**
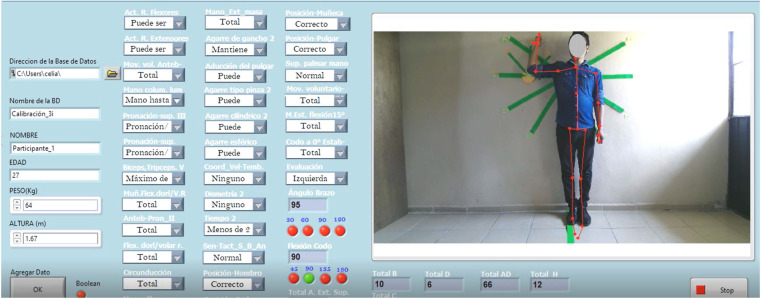
Graphical user interface.

The general participant data, such as name, age, weight, height, shoulder/elbow AROM, and performance level, are registered in the frontal panel of the graphical user interface panel (Figure 1). Thirty control parameters related to upper limb activity covering items I, III, IV, V of section A, B, C, and D of the FMA scoring are also available in this panel; Fugl-Meyer (1975) [28]. These parameters were manually selected by the therapist according to the movements of rotation, pronation, supination, and retraction of the arm, which cannot be acquired by the Kinect v2 sensor, and for activities observed during the test, such as the grip type and hand sensitivity. Section A-II “Voluntary movement within synergies without gravitational assistance”, corresponds to the automatized part of the programming as the shoulder angles in abduction, adduction, flexion, and extension movements of the elbow are acquired through the Kinect v2 sensor. The upper limbs motor performance is shown in the “Total A-D” label. The highest FMA score corresponds to the best motor functioning state of the patient’s limb. An FMA score ranging from 0 to 22 represents no upper limb motor capacity; scores of 23 to 31 represent low capacity; scores of 32 to 47 represent limited capacity; finally, scores from 53 to 66 represent total upper limb capacity [35]. The interface allows visualization of the measurement environment.

**Figure 2:**
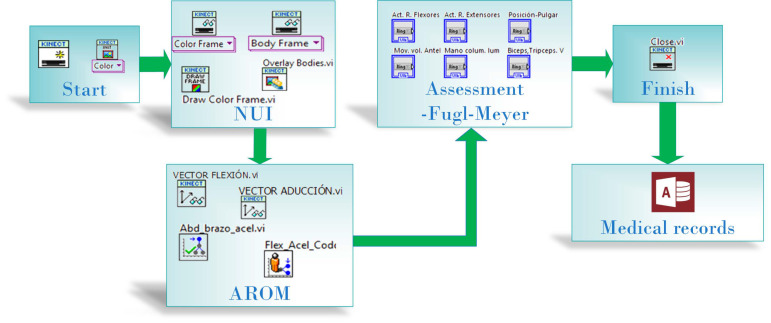
Programming process block diagram.

The graphical user interface was developed using LabVIEW software (14.0, NI, Austin, TX, USA). The Kinect for Windows SDK 2.0 software development kit was used, and MakerHub Interface for Microsoft Kinect One was employed to acquire color, depth, infrared, and sensor skeleton tracking data. The measurement system programming process is shown as a block diagram in Figure 2. In the programming, the motor evaluation is performed by following Fugl-Meyer scale algorithm, where the left and right shoulder and elbow angles are the input data; these vector points are acquired by the Kinect sensor. The process involves the AROM measurement, and the evaluation results are stored in a Microsoft Access database (Microsoft, Redmond, WA, USA).

The AROM calculation of the forward shoulder abduction/adduction in the frontal plane and elbow flexion/extension in the sagittal plane are based on the angle formula between two vectors given by Equation (1) [36]:

(1) $$\mathrm{Cos}\;\theta =\frac{V_{d}\cdot V_{p}}{\left|V_{d}\right|\left|V_{p}\right|}$$

where *θ* is the shoulder/elbow angle, $V_{d}$ is a distal longitudinal vector, and $V_{p}$ is a proximal segment vector.

The segments used for the shoulder angle calculation are summarized in Table 3, selected from the 25 joint points provided by the Kinect v2 sensor [19]. The recommendations established by the International Society of Biomechanics (ISB) were followed [37], but applied only to forearm movements (elbow angle) because of the location of the joint points provided by the sensor.

### 2.3. Experimental Setup

The Kinect v2 sensor (Microsoft, Redmond, WA, USA) was placed on a uniform base at a height of 80 cm and at a distance of 2 m from the participant according to the recommendation of [21]. The Kinect v2 sensor was turned on and run for 25 min before starting the assessments as recommended in [38]. Measurements were carried out in a 3 × 3 m space considering the operational measurement range [19,23,39] with an illumination intensity of 73 Lux (lx).

In order to verify the distance and illumination, 30 angle measurements of the arm at 90 degrees located at a distance of 1 m, 2 m, and 3 m were made. The illumination was measured using a Ut382 Digital luxmeter (UNI-T, Hong Kong, China ). Two illumination values were defined by the minimum (7 lx) and maximum (73 lx) values provided by Salenec (Sentul, Ciudad de México, Mexico) power fixed LED lamp, which by a slide dimmer (Leviton México, Ciudad de México, Mexico) power fixed LED dimmable lamp. The obtained measured angles are shown in Table 4, where optimum performance is observed at 2 m for both 7 and 73 lx, with low variability and an angle difference of about ±3°.

**Figure 3:**
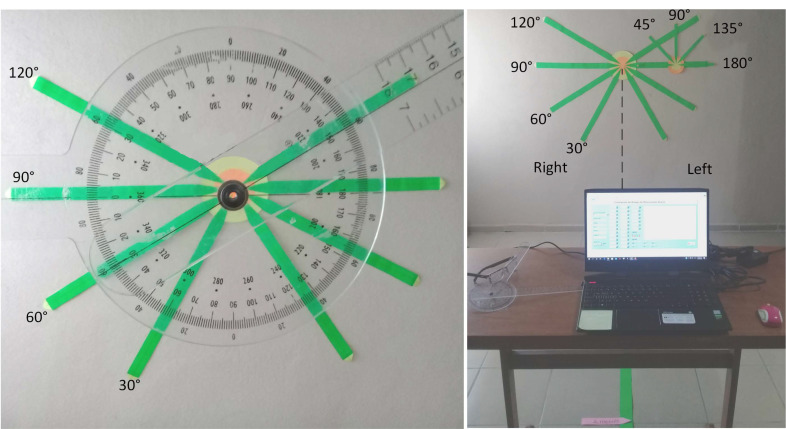
Angle setting.

Fixed angles were marked on a paper background using a universal goniometer (Richardson Products Inc, Gulfstream Rd, Frankfort, KY, USA) as shown in Figure 3. AROM angles were obtained by the Kinect v2 based measurement system. A set of 30 measurements was taken at different angles (30°, 60°, 90°, and 120°) of the left and right shoulder of one participant; also, a set of 30 measurements was taken (45°, 90°, 135°, and 180°) of the elbow of the left and right side, and the standard deviation of the measured angles was ±0.2° on both sides (Table 5).

### 2.4. Statistical Analysis

In order to verify the joint angle differences between those obtained by the Kinect-based measurement system and those obtained by the goniometer, a Wilcoxon rank-sum test equivalent to the Mann–Whitney U-test was carried out for the data resulting from the control and study groups. The statistical analysis was undertaken using MATLAB (R2017b, MathWorks, Natick, MA, USA). The significance level used was α=0.05.

## 3. Results

Table 5 shows the results obtained from the 30 samples using the Kinect at different angles and compared with those using the universal goniometer. The standard deviation of the measured angles was ±0.2° on both sides. The errors obtained are minimal, with an average difference of ±1° between the actual value and the value estimated by the Kinect-based measurement system.

The measurements of elbow flexion and shoulder abduction movement are presented in Table 6. The difference between the angles obtained by both groups was not significant (*p* < 0.05). Similarly, there was no significant difference between the manual FMA measurement and FMA with NUI (*p* < 0.05). The error of the measurement system with NUI is less than ±1°.

**Figure 4:**
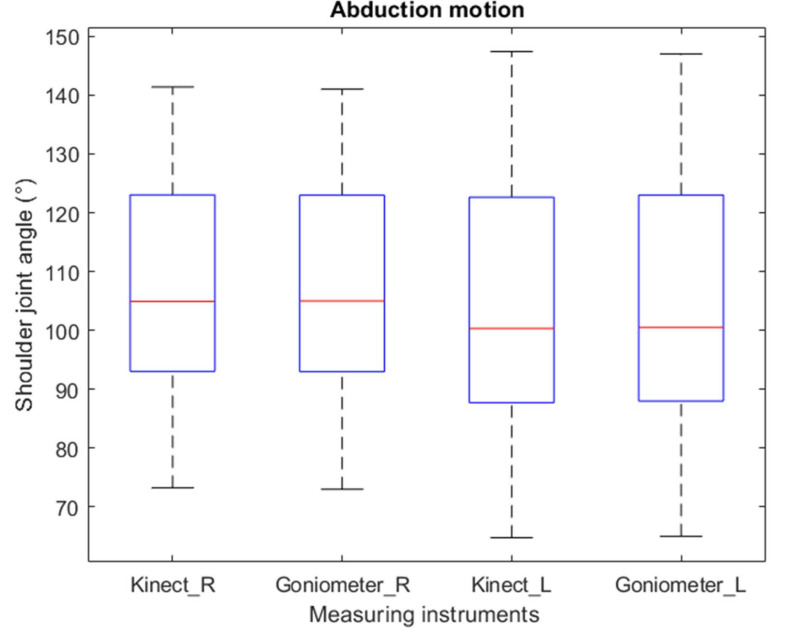
Variability in abduction movement.

The box plot in Figure 4 shows minimal differences between the angular means of the measurements recorded with the Kinect v2 and the goniometer for the right and left limb. As can be observed, the variability between the two devices is not significant. The literal “R” represents the assessment of the right side, and “L” represents the measurements of the left side.

The shoulder abduction and elbow flexion movement measurements for the control and study groups obtained with the NUI system are summarized in Table 7. The difference between the elbow angles obtained in the two groups was not significant (*p* < 0.05). Similarly, there were considerable differences between the FMA and shoulder abduction measurements between the two groups (*p* < 0.05).

**Figure 5:**
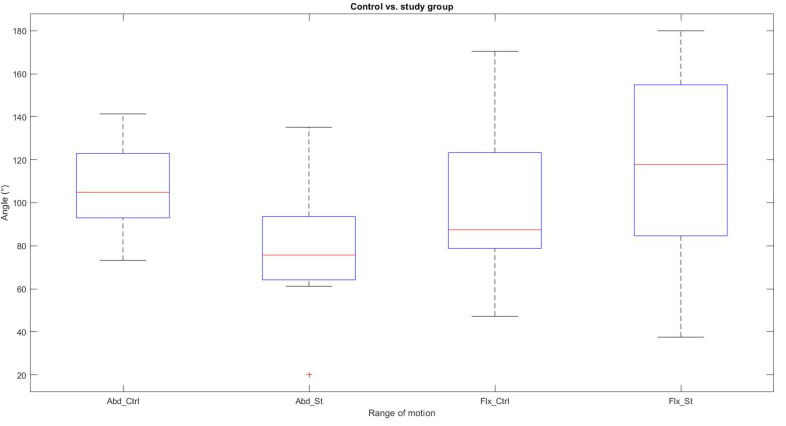
AROM measurements.

The box plot in Figure 5 shows significant differences between the angular means of the abduction movement recorded with the Kinect v2 between the control group and the study group; the study group presents difficulties in reaching a complete AROM in the abduction movement. As can be seen, in the elbow flexion movement, the variability between the two groups is not representative; both groups can flex the elbow. The literal “Abd_Ctrl” represents the abduction movement for the control group, “Abd_St” is the abduction movement in the study group. Similarly, the label “Flx_Ctrl” represents the flexion movement in the control group, and “Flx_St” is the flexion movement in the study group.

**Figure 6:**
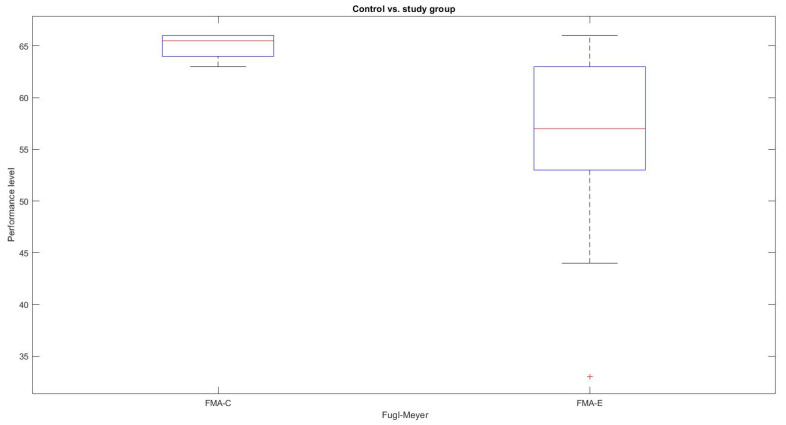
FMA measurements.

Similarly, as shown in Figure 6, according to the FMA scale, the motor performance, of the control group and the study group shows significant variability. The literal “FMA-C” represents the FMA score of the control group, and “FMA-S” is the score of the study group.

## 4. Discussion

The present study enabled angular measurements of the upper limb using the NUI of the Kinect v2 sensor. Data from a universal goniometer were used as a reference to compare upper limb movement analysis measurements.

Concerning the measurements, it is observed in Table 4 that, at one meter, there was a higher variation. The sensor was very close to the participant, and the camera’s field of view did not capture the whole scene, generating flying pixels. Similarly, in the measurement of 3 m, flying pixels were also present. However, the viewing range was higher. Optimum performance was at 2 m at both 7 and 73 lx, showing a minor variability with a difference of ±3°. Our results for distance and height coincide with the work of Cai et al. [21], where they recommend a height of 0.80 cm and a distance of 2 m from the sensor to the participant. However, we added lighting parameters where, according to the characteristics of the infrared camera, RGB camera, and the experiment performed, the proposed system can operate from 7 to 73 lx.

Several studies have shown that upper limb angle measurements based on the Kinect sensor and other reference instruments, such as goniometers or wearable sensors, have a high degree of validity [16,22,40]. The authors Beshara et al. [22] also compared measurements with a goniometer and claimed that the use of wearable inertial sensors in conjunction with Kinect v2 is a reliable and valid way to assess active shoulder flexion and abduction, with minimal measurement errors (2–4°), which represents high reliability. The results of this study show a significant validity of the measurements with the Kinect v2 sensor with an error of ±1° compared to the specialist’s measurements. Our results match the authors’ claims [16] about the reliability of measures with the Kinect sensor, where their study demonstrated an accuracy of ±5°.

Concerning the validity of the measurement system, no significant differences were found (Table 6, *p* > 0.005) between flexion and abduction movements of both shoulder and elbow. Therefore, the AROM measurements of the elbow and shoulder are reliable. Overall, these results indicate that the algorithm programmed with the FMA scale is an effective and alternative tool for determining upper limb motor performance.

By comparison, as shown in Table 7, significant differences were found (*p* > 0.005) between the control group and the study group. The study group, representing a particular case of hemiparesis in the upper extremity, presented a lower motor performance than the control group. A share of 78% showed a total performance, according to the FMA scoring (52–66), 11% a notable capacity (47–52), and 11% a limited capacity (31–47). However, 83% of the cases presented difficulties in performing the activities during the test, mainly in the hand, wrist, and type of grip. Finally, 11% did not achieve a maximum score due to a lack of coordination and speed. This system has the advantage of obtaining angular measurements at 2 m, i.e., without direct contact with the participant. It also semi-automatically measures a level of upper limb motor skills. Unfortunately, in 2017, Kinect v2 sensors (Microsoft, Redmond, WA, USA) were discontinued. However, a viable replacement may be the Azure Kinect (Microsoft, Redmond, WA, USA) [17].

It should be noted that this study was carried out with a small sample size. However, this population was appropriate, particularly when eliminating cognitive and functional deficits that can intervene in the evaluation. The developed assessment system is semi-automatic because it only acquires real-time angles involved in the FMA assessment; the other parameters, such as sensitivity or pain, are entered into the system manually by the specialist. The assessment of motor performance with the FMA scale manually requires approximately 30 min per patient; however, with the semi-automatic method, the time is considerably reduced, to around 5–10 min, depending on the GMFCS and MACS level, and the cognitive level of the patient. For this reason, it is essential to consider these visuomotor factors; otherwise, it can complicate the assessment, as not all patients can follow instructions and perform active movements. Nevertheless, one of the benefits for specialists using the semi-automatic method is quantitative and distance measurement, with less angular measurement error than using a goniometer, as the accuracy of this instrument depends on the experience of use between each evaluator. The implementation of this measurement system is relatively simple, first requiring an exclusive area of 3 × 3 m, with illumination of 7–73 lx, and the relevant equipment; at the time of the evaluation, a distance of 2 m between the sensor and the patient must be considered. Regarding the large-scale implementation of the system, training for technicians is required. For the correct use of the evaluation system, it is also necessary to provide the relevant indications to the specialist or person in charge of the rehabilitation area. This training is left as future work, considering a video tutorial, and providing a system manual for the end-user. It is worth mentioning that the assessment instructions are available at the top of the graphical interface. Similarly, the controls of the latest developed system version indicate the labels of the scale, which a specialist will be familiar with due to familiarity with this type of clinical scale for evaluation of motor performance, i.e., in this case, FMA. Future work will also include Constraint-Induced Movement Therapy (CIMT) in the study group. The results obtained will be used as a basis for comparing measurements before and after the CIMT rehabilitation process to effectively and quantitatively assess progress.

## 5. Conclusions

In both groups, this study showed a significant agreement between the measurements of the angles (abduction and flexion) of the upper limbs (shoulder and elbow) performed with the Kinect v2 sensor and those made manually with a universal goniometer. Our system differed by ±1° compared to the measurements made by the specialist, which represents a small difference. However, the proposed system provides greater accuracy and a more reliable FMA score, allowing the specialist to focus on specific parts of the upper limb for effective rehabilitation.

The proposed system is an effective alternative clinical measurement tool for the analysis of upper limb motor performance. It allows the therapist to analyze the AROM and, at the same time, determine the upper limb motor performance level of the FMA patient. We recommend conducting the evaluations in a controlled area of 3 × 3 m, considering the illumination factor of 73 lx, a distance between the sensor and the participant of 2 m, a sensor height of 0.80 m, and pre-warming of the Kinect, as mentioned above. However, we suggest implementing this evaluation methodology with the available Kinect sensors or with the new versions of Kinect (Azure Kinect (Microsoft, Redmond, WA, USA)).

Finally, the novelty of this system lies in the assessment of AROM and motor performance in children with spastic hemiparesis assisted with the NUI of the Kinect v2 sensor. Tests were conducted with a universal goniometer and supported by clinical scales, such as MACS, GFMCS, FMA, and the Bender Koppitz test. The system also allows distancing strategies to be implemented, i.e., the motor assessment is carried out without direct contact with the patient, thus reducing the probability of infection with the COVID-19 virus.

## Figures and Tables

**Table 1:** Selection criteria.

Control group	Inclusion criteria	Children	Healthy
		Age	4–12 years
		Cognitive profile	Acceptable (Bender Koppitz Test)
		Informed consent	Authorized
Study group	Inclusion criteria	Children	Diagnosis of spastic hemiparesis due to CP
		Age	4–12 years
		Cognitive profile	Acceptable (Bender Koppitz Test)
		GMFCS	Level I–II
		MACS	Level I–II
		Botulinum toxin	Without or more than six months after application
Both groups	Exclusion criteria	Participants with visual, hearing, and severe cognitive impairment.	
	Rejection criteria	Unauthorized informed consent. Participants with general ailments; undergoing pharmacological, medical treatment, and musculoskeletal injuries.	

GMFCS: Gross Motor Function Classification System; MACS: Manual Ability Classification System; CP: cerebral palsy.

**Table 2:** Characteristics of the study population.

CG	P	1	2	3	4	5	6	7	8	9	10	11	12	13	14	15	16	17	18
	FMA	65	64	65	66	66	65	64	66	66	66	63	63	63	63	66	66	66	66
	CA	4	4	5	5	5	6	7	8	8	8	9	9	9	9	11	11	11	12
	MA	4	4	5	5	5	6	7	8	8	8	9	9	9	9	11	11	11	12
SG	FMA	57	60	54	50	63	65	39	64	52	53	57	42	65	65	62	66	31	58
	CA	4	5	5	6	6	8	8	8	9	9	9	9	9	9	10	11	12	12
	MA	-	4	4	4	6	6	5	7	5	-	7	5	8	4	5	7	8	9
	MACS	I	II	I	II	I	I	II	II	II	I	I	II	I	I	I	I	II	II
	GMFCS	II	II	II	II	I	I	II	I	II	II	II	II	I	I	I	II	I	I

CG: control group; SG: study control; P: participants; CA: chronological age; MA: mental age—able to follow instructions.

**Table 3:** Joints for AROM calculation.

Joint	Movement	Segments
Shoulder	Abduction	Hip, Shoulder, Elbow
	Adduction	Hand, Shoulder, Hip
Elbow	Flexion	Shoulder, Elbow, Hand
	Extension	Hand, Elbow y Shoulder

**Table 4:** Variability in distance and illumination.

Illumination 7 lx			
Meters	1 m	2 m	3 m
Mean ± SD	94.19 ±5.64	89.74 ±2.94	89.33 ±4.77
Illumination 73 lx			
Mean ± SD	111.32 ±4.01	91.65 ±2.06	95.96 ±1.96

SD: standard deviation.

**Table 5:** Results of the averages for the right and left limbs.

Assessment	Variable	Degrees °	Mean	Absolute Error	Relative Error (%)
Right	Shoulder (Abduction)	30	30.549	0.549	1.83
		60	59.994	0.005	0.00
		90	90.425	0.425	0.47
		120	120.546	0.546	0.45
		45	45.664	0.664	1.47
	Elbow (Flexion)	90	90.624	0.624	0.69
		135	135.449	0.449	0.33
		180	180.548	0.548	0.30
Left	Shoulder (Abduction)	30	30.257	0.257	0.85
		60	60.590	0.590	0.98
		90	90.514	0.514	0.57
		120	120.876	0.876	0.73
	Elbow (Flexion)	45	45.540	0.540	1.20
		90	90.504	0.504	0.56
		135	135.511	0.511	0.37
		180	180.603	0.603	0.33

**Table 6:** Comparison of joint angles of the upper limb.

Group	Variable	ROM	System	Mean ± SD	H_0_	*p*-Value	
Control	Shoulder abduction	Right AROM	NUI	108.51 ±19.67	0	1	
		Right PROM	Goniometer	108.50 ±19.62			
		Left AROM	NUI	104.01 ±23.44	0	0.98	
		Left PROM	Goniometer	104.05 ±23.44			
	Elbow flexion	Right AROM	NUI	100.85 ±37.88	0	0.98	
		Right PROM	Goniometer	100.77 ±37.88			
		Left AROM	NUI	94.25 ±34.73	0	0.96	
		Left PROM	Goniometer	94.27 ± 34.70			
	FMA		NUI	65 ±1	0		0.12
			Goniometer	65 ±2			
Study	Shoulder abduction	AROM	NUI	81.60 ±26.94	0		0.21
		PROM	Goniometer	81 ±27.57			
	Elbow flexion	AROM	NUI	116.15 ±44.09	0		0.19
		PROM	Goniometer	115.50±43.88			
	FMA		NUI	56 ±10	0		0.78
			Goniometer	56 ±9			

AROM: active range of motion; PROM: passive range of motion; NUI: natural user interface; H_0_ = 0: There are no differences between the measurements of the two instruments.

**Table 7:** Control group vs. study group measurements.

Variable	Group	Mean ± SD	H_0_	*p*-Value
Shoulder abduction	Control	108.51 ±19.67	1	0.0043
	Study	81.60 ±26.94		
Elbow flexion	Control	100.85 ±37.88	0	0.2145
	Study	116.15 ±44.09		
FMA	Control	65 ±1	1	0.0004
	Study	56 ±9		

## Data Availability

Not applicable.

## Abbreviations

AROM	Active range of motion
PROM	Passive range of motion
ROM	Range of motion
FMA	Fugl-Meyer Assessment
NUI	Natural user interface
